# Efficacy and safety of Ex-PRESS® mini shunt surgery versus trabeculectomy for neovascular glaucoma: a retrospective comparative study

**DOI:** 10.1186/s12886-019-1083-4

**Published:** 2019-03-12

**Authors:** Kazuyuki Kawabata, Kohei Shobayashi, Keiichiro Iwao, Eri Takahashi, Hidenobu Tanihara, Toshihiro Inoue

**Affiliations:** Department of Ophthalmology, Faculty of Life Sciences, Kumamoto University, 1-1-1 Honjo, Kumamoto, 860-8556 Japan

**Keywords:** Neovascular glaucoma, Trabeculectomy, Ex-press mini shunt, Efficacy, Safety

## Abstract

**Background:**

The objective of this study is to evaluate and compare the short-term efficacy and safety of Ex-PRESS® mini shunt surgery and trabeculectomy for neovascular glaucoma (NVG).

**Methods:**

Patients with NVG who underwent Ex-PRESS® mini shunt surgery or trabeculectomy as a primary glaucoma surgery between March 2013 and October 2015 were included in the study, and their medical charts were retrospectively reviewed. The Ex-PRESS® and trabeculectomy groups included 14 eyes and 30 eyes, respectively. Surgical failure was defined by an intraocular pressure (IOP) of ≥21 mmHg (condition A) or ≥ 18 mmHg (condition B); Kaplan–Meier survival analyses and the multivariable Cox proportional hazards model were used to assess efficacies.

**Results:**

Kaplan–Meier survival analyses indicated that the probabilities of success at 1 year for the Ex-PRESS® group were 25.7 and 31.8% based on complete and qualified success under condition A, respectively. The corresponding values for the trabeculectomy group were 47.8 and 69.3%, and there was a significant difference in qualified success with condition A (Fig. 1; *P* = 0.018), while there were no significant differences in the other criteria. Ex-PRESS® mini shunt surgery and higher intraocular pressure were independent prognostic factors using Cox proportional hazards model analyses in qualified success as in condition A (*P* = 0.012 and 0.0495, respectively). The occurrences of postsurgical hyphema and bleb leaks were significantly higher in the trabeculectomy group (*P* = 0.005 and 0.008, respectively).

**Conclusion:**

During a 1 year follow-up, Ex-PRESS® mini shunt surgery was a less effective, but safer treatment for NVG compared with trabeculectomy.

## Background

Filtration surgery, including trabeculectomy and tube shunt surgery using glaucoma drainage devices (GDDs), is the surgical method used most commonly to lower intraocular pressure (IOP) in glaucoma patients [1, 2]. Of the various filtration surgeries, the Ex-PRESS® device is unique in terms of being short and plateless and being made of stainless steel. Its surgical procedure is more similar to trabeculectomy, involving a filtering bleb around the scleral flap, compared with tube shunt surgeries using GDDs, such as the Baerveldt, Ahmed, and Molteno tubes. The wound healing process and surgical results of Ex-PRESS® mini shunt surgery have been comparable to those of trabeculectomy in randomized controlled trials, mainly involving primary open angle glaucoma (POAG) patients [3–7]. However, the efficacy and safety of Ex-PRESS® mini shunt surgery for other types of glaucoma, including neovascular glaucoma (NVG), remain unclear.

NVG is a type of glaucoma that is highly resistant to surgical treatment compared with POAG, probably because NVG patients are relatively young, have more histories of intraocular surgeries, and present chronic microinflammation [8–11]. In addition, our recent study reported that the aqueous humor of NVG patients included higher levels of proinflammatory cytokines, such as interleukin (IL)-6, IL-8, monocyte chemotactic protein (MCP)-1, tumor necrosis factor-α, and platelet-derived growth factor-AA compared with both cataract patients and POAG patients [12]. Thus, the process of wound healing after filtering surgery differs between NVG and POAG patients. Here we report a retrospective comparison of the efficacy and safety between trabeculectomy and Ex-PRESS® mini shunt surgery for NVG patients.

## Methods

### Patients

Before analyses, all patient information were anonymized by a researcher independent of the authors. All patients with NVG who underwent Ex-PRESS® mini shunt surgery or trabeculectomy as a primary glaucoma surgery at Kumamoto University Hospital between March 2013 and September 2015 were included, and their medical charts were retrospectively reviewed. Patients who underwent simultaneous procedures of cataract/vitreous and glaucoma surgeries were excluded. When both eyes of a subject fulfilled the inclusion criteria, only the eye treated first during the period of our analyses was included. This retrospective study was approved by the Institutional Review Board of Kumamoto University. All procedures conformed to the Declaration of Helsinki.

### Pre-surgical treatment

Panretinal photocoagulation was completed for each case before surgery. When rubeosis iridis was prominent, 1.25 mg of anti-VEGF antibody (Avastin®, Chugai Pharmaceutical, Japan) was injected into vitreous within 3 days before surgery.

### Surgical methods and management after surgery

We performed trabeculectomy and management after surgery as previously described [13] by experienced surgeons (TI, KI, HT): all surgeons conducted both Ex-PRESS® mini shunt surgery and trabeculectomy. Briefly, a fornix-based conjunctival flap and a triangle half-layer scleral flap were made, and then exposed to mitomycin C (MMC) at 0.04% for 4 min. MMC was washed with balanced salt solution (200 mL). A corneoscleral block was cut out, and a peripheral iridectomy was conducted. The scleral flap and conjunctival flap were closed using 10–0 nylon sutures. After surgery, all patients received similar medical regimens of 1% (*w*/*v*) 0.1% (w/v) topical antibiotics and topical betamethasone for approximately 3 months and topical atropine sulfate for 1 week. In most cases, laser suture lysis was performed when IOP was 12 mmHg or more after surgery. The surgeons who conducted the primary trabeculectomies determined the necessity of postoperative glaucoma eye drops based on the extent of visual field disturbances, the IOP values, and the of bleb appearance by slit lamp biomicroscopy. When the IOPs could not be lowered enough after surgery even using the maximum permissible number of eye drops, additional glaucoma surgeries were conducted.

In Ex-PRESS® mini shunt surgery, a fornix-based conjunctival flap and a trapezoidal half-layer scleral flap were made, and then they were exposed to MMC at 0.04% for 4 min. MMC were washed by balanced salt solution (200 mL). A 25-gauge needle was inserted into the anterior chamber from the surgical limbus underneath the scleral flap. Then the Ex-PRESS® shunt device (Alcon Japan, Tokyo, Japan) was placed in the anterior chamber through the ostium created by the needle, following the manufacturer’s protocol. After fixation of the Ex-PRESS® device, the scleral flap and conjunctival incision were sutured with 10–0 nylon sutures. Postoperative management was the same as in the trabeculectomy described above.

### Main outcome measures

According to the guidelines from the World Glaucoma Association [14], we defined surgical success as an IOP of < 21 mmHg (condition A) or < 18 mmHg (condition B) with (qualified success) or without (complete success) the use of topical ocular hypotensive medication. Follow-up was censored upon the performance of any additional non-glaucoma related surgery, such as vitrectomy, except for phacoemulsification. Complete failure was defined as a requirement for additional glaucoma surgery (except needling procedures) and hypotony of < 4 mmHg. For this definition of surgical failure, the IOP obtained ≥2 months after trabeculectomy was used to avoid the effect of short-term postoperative IOP fluctuations. IOP levels were measured with a Goldmann applanation tonometer. The preoperative IOP for each eye was determined as the average of three values measured during 3 consecutive visits within 1 month before trabeculectomy. For patients who needed the anti-VEGF management, the IOP after the procedure was not included in the preoperational IOP.

### Statistical methods

Data were analyzed using the JMP, version 8 statistical software package (SAS Institute, Cary, NC, USA). An unpaired two-tailed *t*-test, Wilcoxon rank-sum test, and chi-square test were used to compare characteristics and occurrence ratios of surgical complications between the two groups. Kaplan–Meier survival curve analyses were used to compare surgical results between groups with *P*-values derived from the log-rank test. Univariable analyses using the Cox proportional hazards model were used to identify potential prognostic factors, and a factor with *P* < 0.1 was further analyzed by multivariable analyses. A P-value of < 0.05 was considered statistically significant.

## Results

**Fig. 1:**
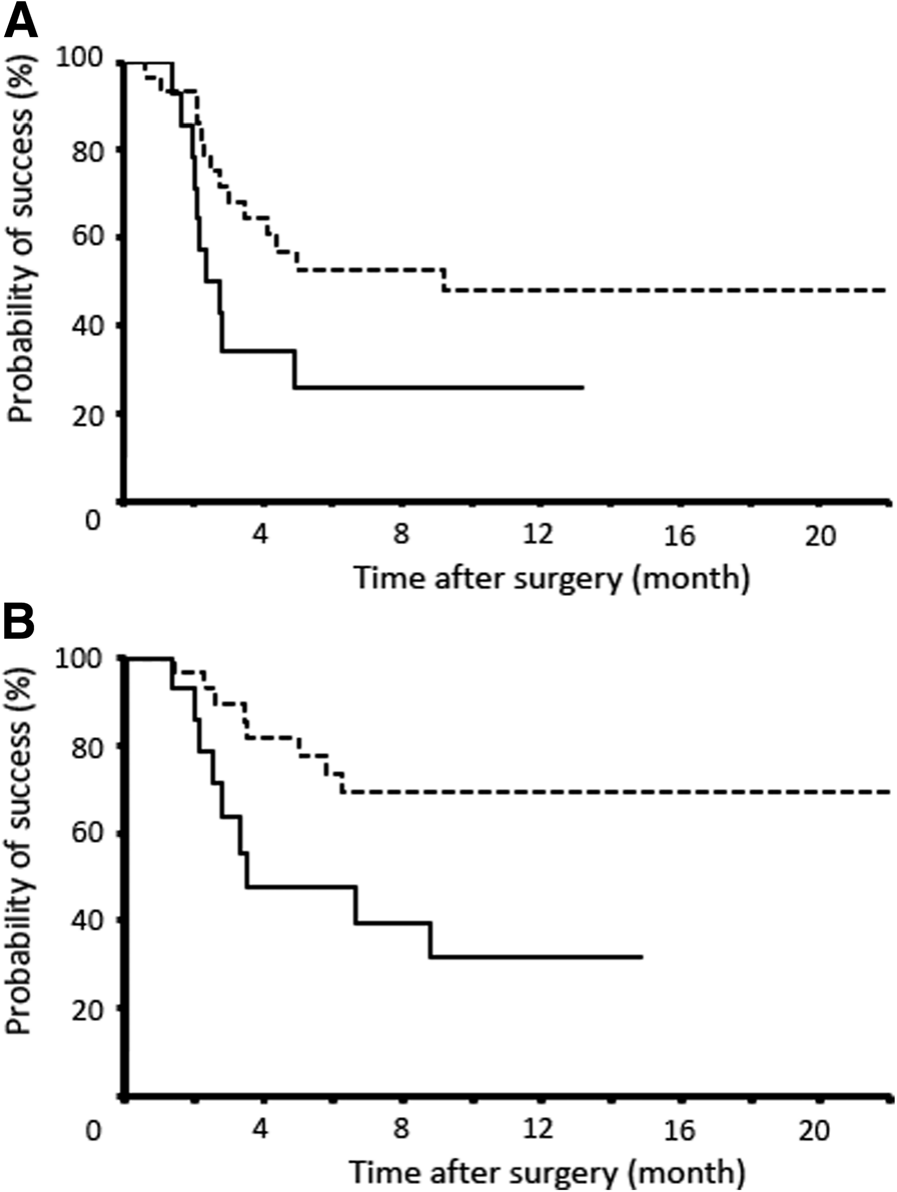
Kaplan–Meier survival plot based on intraocular pressure (IOP) ≥ 21 mmHg without (**a**, complete success) or with (**b**, qualified success) glaucoma eye drops for the Ex-PRESS® group (14 patients, solid line) and the trabeculectomy group (30 patients, dashed line). The probability of qualified success of trabeculectomy was significantly higher than that of Ex-PRESS® (*P* = 0.018)

The Ex-PRESS® group and the trabeculectomy group included 14 eyes and 30 eyes, respectively. Ex-PRESS mini shunt surgery was intensively conducted between March and September in 2014. Trabeculectomy was conducted mainly before and after that duration. Patients’ characteristics are shown in Table 1. There was no significant difference between the groups. Causative diseases in the Ex-PRESS® group were diabetic retinopathy in 10 eyes (71.4%), and central retinal vein occlusion in 4 eyes (28.6%). The corresponding values in the trabeculectomy group were 24 eyes (80.0%) and 2 eyes (6.7%). The numbers of cases with rubeosis iridis were 10 (71.4%) and 20 (66.7%) in the Ex-PRESS® group and the trabeculectomy group, respectively. Kaplan–Meier survival analyses indicated that the probabilities of success at 1 year for the Ex-PRESS® group were 25.7 and 31.8% based on complete and qualified success under condition A, respectively. However, the corresponding values for the trabeculectomy group were 47.8 and 69.3%, and there was a significant difference in qualified success with condition A (Fig. 1; *P* = 0.018), while there were no significant differences in the other criteria for surgical success. Univariable analyses using the Cox proportional hazards model identified Ex-PRESS® mini shunt surgery, younger age, higher preoperative IOP, and avitreous status as significant prognostic factors for surgical failure in qualified success for condition A (Table 2; *P* = 0.027, 0.041, 0.011 and 0.042, respectively). Subsequent multivariable analyses confirmed that Ex-PRESS® mini shunt surgery and higher preoperative IOP were independent prognostic factors for surgical failure (Table 3; *P* = 0.012 and 0.0495, respectively). Needling bleb revision was conducted in four eyes (28.6%) and six eyes (20.0%) in the Ex-PRESS® group and the trabeculectomy group, respectively. The ratios between these two groups were not significantly different (*P* = 0.527).

**Table 1:** Baseline Characteristics of All Patients

Characteristic	Ex-PRESS	Trabeculectomy	*P* value
Number of patients	14	30	–
Male/Female	10/4	25/5	0.362
Age (years)			
Mean ± SD	62.8 ± 17.4	62.6 ± 15.3	0.960
Range	29 to 88	29 to 93	
Preoperative IOP (mmHg)			
Mean ± SD	37.4 ± 9.7	35.4 ± 8.9	0.487
Range	21.0 to 57.3	18.7 to 51.3	
Number of preoperative glaucoma drops			
Mean ± SD	4.0 ± 0.4	4.2 ± 0.5	0.301
Range	3 to 5	3 to 5	
Follow-up period (months)			
Mean ± SD	15.7 ± 5.9	15.6 ± 12.4	0.987
Range	2.8 to 25.1	0.3 to 24.4	
Causative disease of NVG			0.070
Diabetic retinopathy	10 (71.4%)	24 (80.0%)	
CRVO	4 (28.6%)	2 (6.7%)	
Others	0	4 (13.3%)	
Oclular findings			
Rubeosis iridis	10 (71.4%)	20 (66.7%)	0.752
History of previous ocular surgery			
IVB injection within 3 days before surgery	11 (78.6%)	18 (60.0%)	0.226
Cataract surgery	8 (57.1%)	17 (56.7%)	0.976
Vitrectomy	7 (50.0%)	13 (43.3%)	0.679

Abbereviations: *CRVO* central retinal vein occlusion, *IOP* intraocular pressure, *IVB* intravitreal bevacizmab, *SD* standard deviation

**Table 2:** Results of univariable analysis using the Cox proportional hazards model for Condition A (qualified success)

Variable	RR	95% CI	*P* value^a^
Ex-PRESS procedure (vs trab)	2.99	0.07 to 1.04	0.027
Age (per year)	0.97	−0.066 to − 0.001	0.041
Male (vs female)	0.84	−1.21 to −1.10	0.768
Preoperative IOP (per mm Hg)	1.08	0.017 to 0.13	0.011
Rubeosis iridis	1.47	−0.61 to 1.53	0.464
Pseudophakia	1.35	−0.67 to 1.37	0.547
Avitreous status	2.73	0.034 to 2.07	0.042
History of IVB within 3 days before surgery	1.44	−0.63 to 1.51	0.487

*CI* confidence interval, *IOP* intraocular pressure, *RR* relative risk, *trab* trabeculectomy, *vs* versus

^a^The *P* values were determined by means of the Wald test in the Cox proportional hazardsmodel. Significant at *P* < .05

**Table 3:** Results of multivariable analysis using the Cox proportional hazards model for Condition A (qualified success)

Variable	RR	95% CI	*P* value^a^
Ex-PRESS procedure (vs trab)	3.96	0.15 to 1.26	0.012
Preoperative IOP (per mm Hg)	1.06	0.00014 to 0.12	0.0495
Age (per year)	0.969	−0.078 to 0.015	0.185
Avitreous status	1.38	−0.96 to 1.68	0.629

*CI* confidence interval, *IOP* intraocular pressure, *RR* relative risk, *trab* trabeculectomy, *vs* versus

^a^The *P* values were determined by means of the Wald test in the Cox proportional hazardsmodel. Significant at *P* < .05

When comparing the incidence ratios of postsurgical complications between the groups, the ratios of hyphema and bleb leaks were significantly higher in the trabeculectomy group (*P* = 0.005 and 0.008, respectively; Table 4). However, the incidence ratios of choroidal detachment or shallow chamber were not different between the groups. No case of bleb-related infection, suprachoroidal hemorrhage, or tube dislocation was observed during the observational period.

**Table 4:** Comparison of Incidence Ratio of Postoperative Complications

Complication	Ex-PRESS	Trabeculectomy	*P* value*
Hyphema	3 (21.4%)	20 (66.7%)	0.005
Bleb leakage	2 (14.3%)	17 (56.7%)	0.008
Choroidal detachment	2 (14.3%)	4 (13.3%)	0.932
Shallow chamber	0	3 (10.0%)	0.220
Bleb-related infection	0	0	N/A
Suprachoroidal hemorrhage	0	0	N/A
Tube dislocation	0	N/A	N/A

*N/A* not applicable; *Chi square test

## Discussion

We compared the 1 year surgical results of Ex-PRESS® mini shunt surgery with trabeculectomy for NVG patients. Since, past studies about the results of Ex-PRESS® mini shunt surgery for NVG included less than 10 patients [15, 16], or non-comparative one [17], this is the first report of such a comparison for NVG, and therefore should have important relevance to current clinical practice.

Past studies of the surgical results of Ex-PRESS® mini shunt surgery for POAG patients have indicated similar efficacies for IOP reduction and less frequency of surgical complications [3–7, 18–20] when compared with alternative procedures. However, the present study shows that the probability of success of Ex-PRESS® mini shunt surgery was significantly lower compared with trabeculectomy in qualified success with condition A (*P* = 0.005). Furthermore, subsequent multivariable analyses using the Cox proportional hazards model confirmed that Ex-PRESS® tube shut surgery was an independent prognostic factor for surgical failure in the same condition (*P* = 0.001), indicating that Ex-PRESS® mini shunt surgery was less effective in lowering IOP compared with trabeculectomy. This difference in the efficacies of this surgery between NVG and POAG patients is possibly due to differences in wound scarring, which is more excessive in eyes with NVG than in those with POAG. The aqueous humor in eyes with NVG contains more proinflammatory cytokines, such as IL-6, IL-8, and MCP-1, compared with POAG, leading to enhanced inflammatory reactions after ocular surgery [12]. Though past studies reported that wound healing after Ex-PRESS® mini shunt surgery was similar to that after trabeculectomy using an animal model [21], there may be differences in the processes between the surgeries in eyes with NVG. Notably, there may be less outflow volume of aqueous humor into the filtering bleb at the early postsurgical phase after Ex-PRESS® mini shunt surgery. This possibility was consistent with a lower frequency of shallow chambers as a postsurgical complication compared with trabeculectomy. For high risk eyes, a relatively large amount of aqueous outflow into the bleb at the early postsurgical phase may be required to control IOP.

In the present study, the frequencies of hyphema and bleb leaks were lower in the Ex-PRESS® group compared to the trabeculectomy group (*P* = 0.005 and 0.008, respectively). The advantages over conventional trabeculectomy would be omissions of sclerostomy and peripheral iridectomy, and these features must reduce the risk for hyphema. These results are at least partially consistent with past studies reporting that Ex-PRESS® mini shunt surgery reduced the risk for postsurgical hyphema in eyes with POAG [6, 15, 19]. Because NVG itself is a risk factor for hyphema after trabeculectomy [22], the advantage of Ex-PRESS® mini shunt surgery over trabeculectomy regarding the risk for postsurgical hyphema may be enhanced in NVG cases. However, the risk for bleb leaks might be explained by less outflow volume of aqueous humor into the filtering bleb during the early postsurgical phase. Because the difference in the frequency of bleb leaks has not been reported in past studies for POAG cases, further studies are required to definitively establish the risk for bleb leaks in high risk cases. The bleb leakage ratio was 56.7% in trabeculectomy group. The ratio is higher compared to previous studies. A possible explanation for this difference may be that all cases underwent filtration surgery by making fornix-based conjunctival flap, which is known to have more bleb leak compared to that by making limbal-based conjunctival flap.

Univariable analyses using the Cox proportional hazards model depicted younger age, higher preoperative IOP, and avitreous status were prognostic factors. These results are consistent with our previous study on the surgical results of trabeculectomy of 101 NVG patients, in which younger age and previous vitrectomy were prognostic factors for surgical success [9]. However, multivariable analyses showed that preoperative IOP, not younger age or avitreous study, was an independent prognostic factor for surgical success in NVG cases (*P* = 0.0495). Since the patients are different and the methods of statistical analyses are not identical with previous reports [23–25], and the sample number in the present study was not large, independent risk factors should be further investigated in future.

The limitations of the present study include the retrospective manner and our relatively small sample size as described above. In addition, the follow-up period may not be enough to predict the long-term outcomes, since the pathological progression of glaucoma requires decades in many cases. Thus, the data should be interpreted with caution. Larger-scale randomized studies are required for the conclusive data.

## Conclusion

In a 1-year follow-up, Ex-PRESS® mini shunt surgery was less effective, but a safer procedure than trabeculectomy, for treatment of NVG patients.

## Abbreviations

GDD: Glaucoma drainage device
IL: Interleukin
IOP: Intraocular pressure
MCP: Monocyte chemoattractant protein
MMC: Mitomycin C
NVG: Neovascular glaucoma
POAG: Primary open angle glaucoma
